# Whole blood biophysical immune profiling of newborn infants correlates with immune responses

**DOI:** 10.1038/s41390-025-03952-y

**Published:** 2025-03-31

**Authors:** Kerwin Kwek Zeming, Genevieve Llanora, Kaiyun Quek, Chin Ren Goh, Nicholas Zhi Heng Ng, Jongyoon Han, Kee Thai Yeo

**Affiliations:** 1 Critical Analytics for Manufacturing of Personalized Medicine, Singapore-MIT Alliance for Research and Technology, Singapore, Singapore; 2 Department of Neonatology, KK Women’s & Children’s Hospital, Singapore, Singapore; 3 Department of Biological Engineering, Massachusetts Institute of Technology, Cambridge, MA USA; 4 Department of Electrical Engineering, Massachusetts Institute of Technology, Cambridge, MA USA; 5 Antimicrobial Resistance, Singapore-MIT Alliance for Research and Technology, Singapore, Singapore; 6 Duke-NUS Medical School, Singapore, Singapore; 7 Yong Loo Lin School of Medicine, National University of Singapore, Singapore, Singapore

## Abstract

**Background:**

There is a current, absence of reliable, blood-sparing, diagnostic tools to measure and trend real-time changes in the levels of inflammation and its effects on the immune cells in the infant.

**Methods:**

We deployed the BiophysicaL Immune Profiling for Infants (BLIPI) system in the neonatal intensive care unit to describe immune cell biophysical profiles using 50 microliters of blood per sample from term and preterm infants.

**Results:**

A total of 19 infants (8 term, 11 preterm) were recruited and 24 blood samples were collected in their first month. Based on the profiles of immune cells’ size and deformation, there was a clear distinction between term and preterm infants, with 48/50 markers significantly different. A preterm infant with late-onset bacterial sepsis had notable size and deformability differences compared to the rest of the preterm cohort. There was a significant correlation between immune cell biophysical profiles and clinical markers such as C-reactive protein, white blood cell counts, and immature-to-total neutrophil (I:T) ratios, with Pearson correlation coefficients for linear regression models of 0.98, 0.97 and 0.94 respectively.

**Conclusion:**

This study highlights the potential for the biophysical immune cell profiling system to provide an overview of the infant’s current immune activation and response.

**Impact:**

- We present a novel, minimally invasive diagnostic system that leverages the physical properties of immune cells to provide a rapid and direct assessment of the immune status, requiring 20 times less blood volume than standard tests.
- This study demonstrates the potential of a compact, deployable system that is capable of performing biophysical profiling to assess immune cell activation in term and preterm infants, by revealing distinct differences in cell size and deformation between groups.
- The system’s sensitive, quantitative measures were correlated with routine clinical biomarkers, highlighting its ability to provide a rapid, minimally invasive, real-time monitoring of neonatal immune status.

## Introduction

Prolonged, unregulated inflammation during the perinatal and neonatal periods often leads to severe complications for the infant. Pregnancy-related conditions such as chorioamnionitis, infections, preeclampsia, and metabolic syndrome contribute to the establishment of a pro-inflammatory fetal milieu.^1,2^ After birth, factors such as hypoxia/hyperoxia, meconium aspiration syndrome, and sepsis exacerbates this condition for the infant.^3^ The inherent immaturity of the neonatal immune and organ systems amplifies their underlying vulnerability to inflammation.^4,5^ Premature infants are particularly at risk, contributing to the development of morbidities like bronchopulmonary dysplasia (BPD),^6,7^ necrotizing enterocolitis (NEC),^8^ retinopathy of prematurity (ROP),^9^ and neurodevelopmental impairments.^10^ Effective management of inflammatory triggers is crucial for improving infant outcomes and reducing morbidities.^11–14^

The current absence of a reliable blood-sparing diagnostic tool to measure and trend real-time changes in the levels of inflammation and its effects on the immune cells in the infant prevents the early detection and monitoring of inflammatory conditions in this vulnerable group. C-reactive protein (CRP), procalcitonin (PCT) and full blood count (FBC) indices are used in clinical care to ascertain the risk of infection and inflammation in the newborn period, although these do suffer from a lack of specificity and sensitivity.^15,16^ These markers can be elevated in a wide range of conditions including infectious and non-infectious states.^17^ Additionally, the requirement for a significant blood volume for each test (approximately 1–2% of the total blood volume in very preterm infants) can hinder our ability to continuously track these results over time and may lead to complications such as anaemia.

These diagnostic gaps underscore an urgent need for innovative solutions tailored to address this deficiency in neonatal care, allowing for timely clinical decision-making and mitigation of potential complications. In this proof-of-concept study, we explore the utility of biophysical immune response measurements of the neonatal immune cells, in order to address the unmet needs highlighted. This novel and minimally invasive diagnostic technology capitalizes on the physical characteristics of immune cells, providing an expeditious and direct measure of the immune system’s status.

## Methods

### Study population and procedures

Term and preterm infants <32 weeks gestation born at KK Women’s & Children’s Hospital (KKH), Singapore were recruited into the study from 1 March 2022-31July 2023. KKH is the a tertiary-level perinatal centre with up to 12,000 births annually. Infants with known major congenital conditions or chromosomal anomalies were excluded from this study. We deployed the BiophysicaL Immune Profiling for Infants (BLIPI) system in the KKH Neonatal Intensive Care Unit (NICU) in January 2022. Training of staff members on the study workflow and usage of the system occurred over a 3-month period and recruitment of participants commenced thereafter. Optimization of the BLIPI system and of the sampling workflow continued through the recruitment period, with intermittent periods of active recruitment. Blood samples from term infants, born of uncomplicated pregnancies in the delivery suite, were obtained from leftover cord blood samples which are routinely collected for clinical testing. As peripheral blood sampling from well, term infants are not routinely performed, we were unable to perform comparisons of the biophysical profile from the peripheral samples for this current study. Preterm arterial blood samples for this study was obtained during routine blood taking by the NICU team. Demographic and clinical data were obtained from consented participants. Laboratory test results from indicated or routinely performed blood tests (FBC, CRP), corresponding to each BLIPI test, were collected. Specifically, data on total white blood cell (WBC) count, immature: total (I:T) neutrophil count and CRP results were included.

For all blood samples, between 30 and 50 microliters of whole blood was mixed with 2 microliters of ethylenediaminetetraacetic acid (EDTA) at a concentration of 0.1 M in a 250 µL Eppendorf tube. A total of 20 microliters of the mixed blood samples was then loaded onto the microfluidic chip using a pipette before initiation of the test. All blood samples were processed and tested within 1 h of sample collection.

### About biophysical immune cell profiling

Cell size and deformability profiling is a minimally invasive diagnostic technology which capitalizes on the physical characteristics of immune cells, providing an expeditious and direct measure of the immune system’s status. This is based on our previous pilot studies profiling blood samples from adult patients in the emergency room setting; albeit with blood drawn, transported and profiling performed in a laboratory setting with a bench microscope.^18^ In this current study, we developed a compact system for real-time immune profiling in the NICU. A disposable silicone polymer test chip was designed for rapid processing of whole blood (50 µL) and profiling of immune response at point-of-care (<15 min).

The BILPI test is based on a pillar array as an “obstacle” course for the immune cell to interact with as they flow within the array (Supplementary Video S1 and S2). This pillar array interacts and sorts immune cells based on size and deformability with a resolvable resolution of ~200 nm. The cells are deflected by the pillars into specific trajectories uniquely inherent to the cell properties of size and deformability, resulting in a histogram distribution of cells at the microfluidic outlet. These histogram signatures are modulated by hydrodynamic flowrates and unique pillar structure (Supplementary Fig. S1). The profiling readout is based on the output of immune cell trajectories corresponding to unique biophysical signature of the sample.

### Deployment of the BLIPI system in NICU

**Fig. 1:**
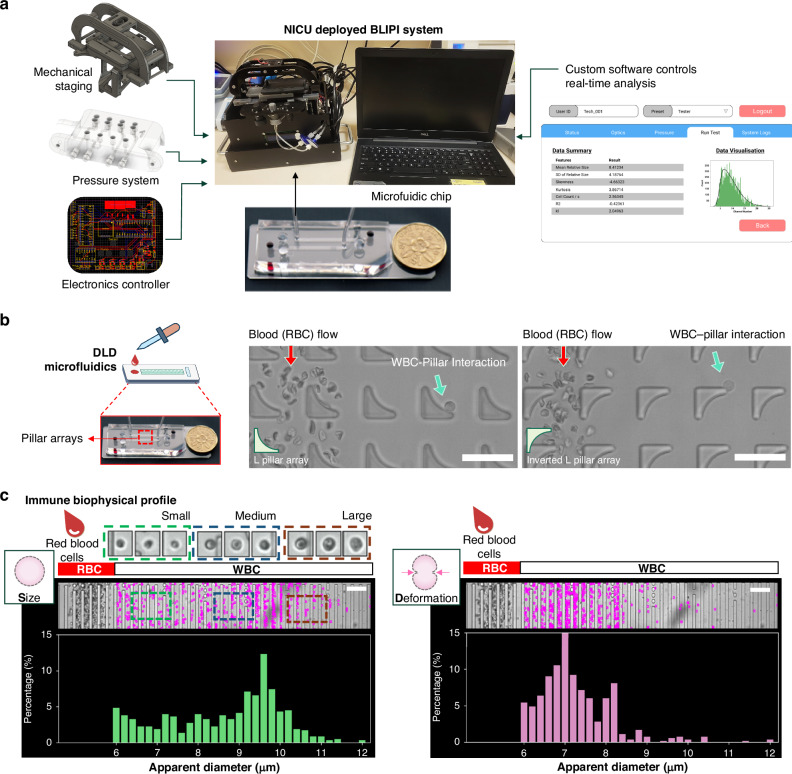
Custom hardware and software for the real-time detection of immune cell biophysical signatures in NICU. **a** Illustrates the functions designed into the system including software and hardware controls. A disposable assay kit was developed for the point-of-care immune profiling test. **b** Shows the workings of the microfluidic device with blood cells (RBCs and WBCs) interacting with the unique pillar shape(s) array. WBC are displaced laterally away from the RBC flow stream and experiencing hydrodynamic forces while interacting with the pillars. Scale bar represents 40 µm. **c** Shows the real-time detection of WBC shown as a pink dot in the image (Supplementary Video S3 and S4). The histogram in the microfluidic chip represents the distribution of immune cell apparent diameter at a given flowrate. Scale bar represents 100 µm.

To enable the biophysical profiling of immune cell within samples in the NICU, a custom microfluidic analysing system was assembled based on 4 key components: (i) microscope and stage holder for the microfluidic chip, (ii) pressure control for fluid and sample handling, (iii) electronics and microcontrollers for communication with a laptop computer and (iv) software control and data analysis using a laptop computer. Individually packed microfluidic test kits comprising microfluidic chip with tubing connectors and pre-loaded buffer reservoirs was prepared for the tests to be performed in the NICU. The compact actuated microscope stage with fluid-pressure regulated module is controlled by an electronic board (Fig. 1a, b). A GUI was designed to interact with the system and controls the immune profiling by the microfluidic chip.

### Biophysical profiling protocol

To initiate the test, the microfluidic chip is placed on the assembled system. The tubings and reservoirs are connected to the microfluidic system. The chip is primed with 2% w/v pluronic F127 solution for 5 min before the chip is ready for biophysical profiling of samples. To operate the BLIPI system, the operator loads a single drop of blood (at least 20microliter) into a well within the chip that has already been primed with fluid. The graphic interface will prompt the operator to start the test after the sample-loaded chip is placed onto the system. As the blood flows through the microfluidic device, a real-time imaging algorithm detects immune cells present and measures their distribution profile at the detection region of the chip. (Fig. 1c). Data generated from the BLIPI study was saved and analysed on the connected laptop computer.

**Fig. 2:**
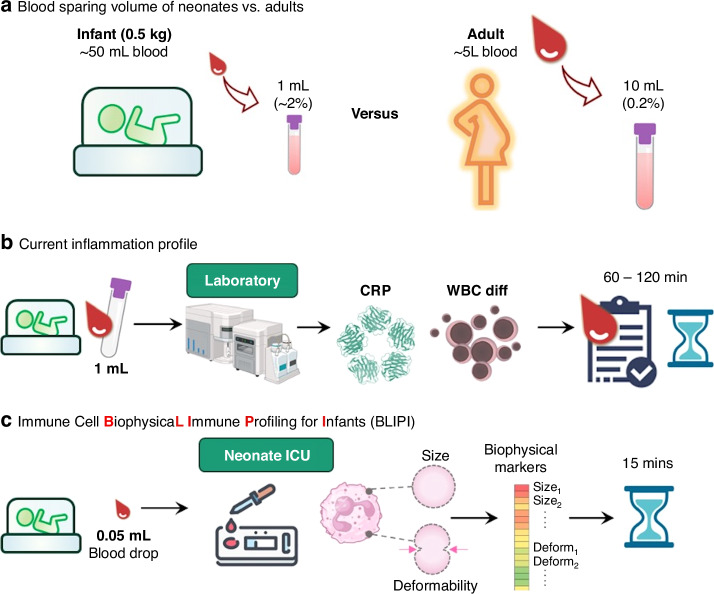
Blood-sparing and real-time measurements using the BLIPI workflow within the neonatal intensive care unit for point-of-care measurement of host immune cell response. **a** Highlights the significant impact of the blood volume required for individual testing for the adults (mother) compared to the infant. **b** Illustrates the current clinical workflow and routine testing done with a minimum of 1 mL of blood drawn in EDTA tubes for hospital laboratory tests. Results are expected within 60–120 min. The BLIPI system processes up to 0.05 mL or 50 µL of blood and measures size and deformability biophysical properties of immune cells within 15 min as shown in (**c**). These raw profiles are processed and fed into feature transform functions to extract useful information for downstream classification and enable measurement of host immune responses. Routine blood tests may require up to 20× more blood volume compared to biophysical profiling in the preterm infant (1 mL compared to 0.05 mL). These blood volume requirements would be compounded in situations which require multiple testing.

The apparent size histogram of the immune biophysical profile range from 6 to 12 µm with bins of 200 nm each, as described in our previous publication.^18^ The microfluidic chip system allows for the measurement of biophysical changes in size and deformability in individual immune cell, with a precision of up to 200 nm. To measure the deformability, increasing the fluid flow velocity increases the hydrodynamic forces on the immune cell which in turn compresses the cell resulting in a downward shift in apparent size histogram seen in Fig. 2c.

### Data analysis

To enable analysis of the biophysical signatures, video frames are captured and processed in real-time using python scripts for cell detection and histogram bin tabulation. The processed data is saved and further analysed to extract biophysical markers using a secondary script that imports the histogram data, applies the transform functions, and generates 50 resulting biophysical features (Supplementary Table S1). Machine classification of the features data were performed using existing modules in python such as pandas, sci-kit learn and SciPy for all data normalization, statistical analysis, correlation analysis, UMAP and hierarchical clustering. The recursive feature elimination methods used in this study is part of the scikit-learn library where its function is to eliminate the lowest contributing features to the performance of the linear regression model until the target number of features is reached. The data visualization and plots were performed using matplotlib and seaborn python modules.

The linear regression model is given by the following equation:

$${y}_{{clinical\; parameter}}={\beta }_{0}+{\beta }_{1}{x}_{1}+{\beta }_{2}{x}_{2}+\ldots +{\beta }_{k}{x}_{k}$$

Where y represents the clinical parameter of CRP, WBC counts or I:T ratio, x represents the BLIPI measured features shown in Table 1, k = 15 represents the feature variables, β represents the linear regression coefficients shown in Table 1.

**Table 1:** Linear regression model coefficients for each clinical parameter.

	CRP		WBC counts		I:T ratio	
k	${\beta }_{k}$	${x}_{k}$	${\beta }_{k}$	${x}_{k}$	${\beta }_{k}$	${x}_{k}$
0	15.42	–	15	–	0.06	–
1	11.5	D37	3.39	D47	−0.024	D47
2	11.68	D46	0.86	S18	−0.019	D26
3	−9.26	S43	−2.68	S19	0.017	S4
4	−2.8	D29	2.79	D49	0.032	D49
5	−4.27	S9	1.36	D46	−0.021	D46
6	−3.46	S2	3.61	D26	0.02	D29
7	−4.6	D14	−2.39	S7	0.012	S43
8	−5.22	S10	0.95	S8	−0.019	S18
9	3.59	S8	4.4	S40	0.003	D33
10	2.55	S0	−2.76	S43	0.016	D14
11	0.64	S15	1.94	S6	0.018	D48
12	5.05	S7	2.34	D38	0.02	S10
13	−6.03	D34	−2.63	S3	0.014	S7
14	3.86	D47	1.73	D37	0.002	S0
15	−4.52	D26	1.53	D33	0.01	D17

k represents the feature variables, β represents the linear regression coefficients, x represents the BLIPI measured features.

### Institutional review board

The study was approved by the Singhealth Centralised Institutional Review Board and informed consent was obtained for all participants (CIRB Ref. No. 2016/2791).

## Results

### Characteristics of study cohort

We recruited a total of 19 infants in this current study – 8 term infants and 11 preterm infants. All term infants were from uncomplicated pregnancies and predominantly singleton births (Table 2). The majority of the preterm infants were of extremely low gestational age, with median gestational age of 24 weeks (IQR 24–25) and birthweight of 650 gm (IQR 590–725) (Supplementary Table S2). A total of 24 blood samples were taken within the first month of life – 8 term cord blood samples and 16 preterm arterial blood samples. Four preterm infants provided multiple blood samples over the course of their participation in this study. Median age at testing for the preterm cohort was 5 days of life (IQR 4,10). Corresponding CRP and full blood counts done within the same day were recorded.

**Table 2:** Clinical characteristics and laboratory parameters of study cohorts (19 infants).

	Preterm cohort 11 infants	Term cohort 8 infants
Median gestational age, weeks (IQR)	24 (24–25)	38 (37–38)
Median birthweight, gm (IQR)	650 (590–725)	2885 (2587–2538)
Median age of testing, days (IQR)	5 (4–10)	At birth
Median CRP at the time of testing, mg/dL (IQR)	7.1 (2.1–11.0)	NA
Median WBC count, ×10^9^/L (IQR)	10.7 (9.0–20.7)	NA
Median Absolute neutrophil count, cells/μL (IQR)	6.1 (4.6–14.9)	NA
Median I:T ratio, (IQR)	0.02 (0.02–0.08)	NA
Number of samples, *n*	16	8

*CRP* C-reactive protein, *I:T ratio* immature to total neutrophil ratio, *WBC* white blood cell.

Figure 2a, b highlights the BLIPI investigation workflow with the recruitment of term and preterm babies, emphasizing the key differences between the point-of-care testing and current routine testing. In the preterm population, each set of routine blood tests of CRP and FBC required up to 20× more blood volume compared to biophysical profiling in the preterm infant (2% vs 0.1% of total blood volume for a 500 gm infant). Additionally, tests result with the BLIPI system were available within 15 min as opposed to typical 60–120 min for central laboratory testing (Fig. 2c).

### Biophysical profiling of blood samples

**Fig. 3:**
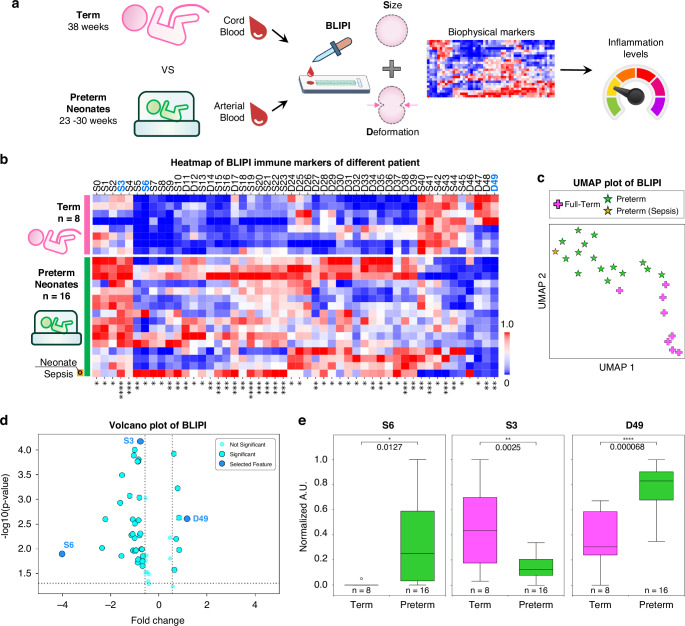
Biophysical profile of immune cells of full-term and preterm babies. **a** Samples from two clinical cohorts corresponding to full term babies (*n* = 8) and preterm neonates (*n* = 16) were profiled using the custom microfluidic assay deployed within the NICU for the comparative profiling of immune biophysical properties. **b** BLIPI immune markers of size (S) and deformability (D) for the two cohorts are shown in a heatmap in a normalized scale from 0 to 1. One preterm infant developed bacterial sepsis and is highlighted in orange. **c** An unsupervised UMAP representation of the BLIPI immune data showing various clusters for full-term and preterm cohort with a single sepsis data point represented in orange. **d** A volcano plot illustrating the fold-change versus *p*-value significance with the dotted line representing boundary of significance. Selected immune profiles of interest are highlighted in darker shade of blue and labelled. **e** The labelled BLIPI markers are shown in box plots with the normalized auxiliary unit values. An independent two-tailed Student *t* test was performed for the two cohorts with *p* < 0.05, 0.01, 0.001 and 0.001 represented by *, **, *** and **** respectively.

To determine the baseline characteristics of the biophysical measurement of blood 8 samples from term (cord blood) and 16 preterm samples (arterial peripheral blood), we sampled and analysed data obtained from both cohorts (Fig. 3a and Supplementary Table S1). From the different profiles of size and deformation of the immune cells measured, there is a clear distinction between the two cohorts as illustrated in the heatmap (Fig. 3b). Out of the 50 biophysical markers, 48 (98%) were significantly different between the two groups. A complete list and description on the biophysical markers evaluated can be found in Supplementary Table S1.

Among the preterm infants profiled, one infant developed late onset sepsis (Fig. 3b, labelled in orange). We noted significant differences in the size and deformability profiles as compared to the rest of the preterm group highlighted in Fig. 3b. Distinct clustering of the different comparator’s groups was noted based on unsupervised UMAP plot of all the biophysical profiles measured (Fig. 3c). Selected biomarkers, S6 (histogram bin value 31), S3 (average histogram value) and D49 (Kurtosis of deformed cell profile) were highlighted in a volcano plot showing both significance levels and fold changes in expression between the two cohorts (Fig. 3d, e). S6 and S3 immune cells measurements show an increase for preterm infants while D49 deformability measurements were significantly decreased.

### Biophysical profiling and correlation with routine tests

**Fig. 4:**
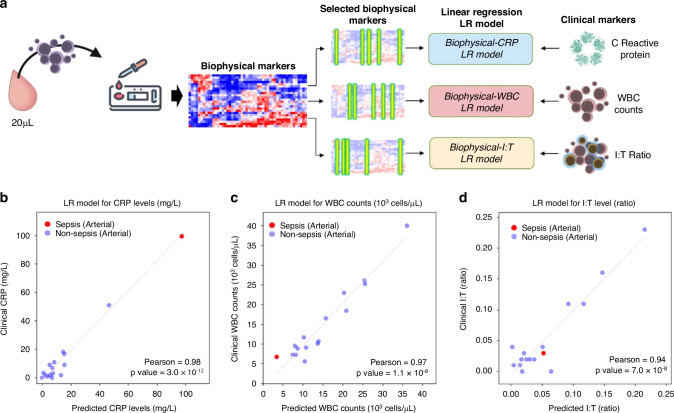
Linear regression (LR) models correlating biophysical and clinical markers. The schematic of the LR models developed based on selected biophysical markers correlating immune cell inherent properties to clinical indications such as C-reactive protein levels, WBC counts and Immature-to-total WBC count (I:T) ratios (**a**). The blind prediction of clinical markers based on the LR models showing strong Pearsons correlation with *p*-values < 0.001 for CRP levels (**b**), WBC counts (**c**) and I:T ratios (**d**).

Given that neonates are inherently vulnerable to inflammatory responses and immune dysregulation, we performed correlations with routinely-used clinical markers of inflammation and immune activation (Fig. 4a). Three indicators, namely CRP, white blood cell (WBC) counts and Immature to Total neutrophil (I:T) ratios, were measured in our central laboratory. Features selection using recursive feature elimination and linear regression method was performed to develop a model for linear correlation between biophysical immune profiling with these conventional immune indicators. The algorithm for the modelling can be seen in Supplementary Figs. S2–S4. The linear regression model performance for CRP, WBC and I:T based on the number of biophysical features used for linear regression model is shown in Supplementary Fig. S5.

To minimise overfitting, a split-test-validate algorithm was used. Briefly, the samples were used for a sandboxing split train-test of 4:1 ratio to develop a linear regression model (Supplementary Fig. S4). The model is then validated by predicting the clinical test data value of the sample that was removed during the train-test modelling. This algorithm was iteratively performed for all samples, resulting in the three plots seen in Fig. 4b–d. Among the samples, the sepsis neonate is highlighted as a red data point. The Pearson correlation coefficient for the linear regression models are 0.98, 0.97 and 0.94 for CRP, WBC counts and I:T ratios. Interestingly, the preterm infant with Gram negative sepsis had a high CRP of ~100 ug/mL with the lowest WBC counts in the cohort. It is important to note that meaningful data was obtained from a single drop of blood, no more than 50 microliter, with strong correlation values with routinely-used immune/inflammatory markers. This indicates that potential for a single biophysical measurement through the BLIPI system to provide a systemic profile of the infant’s current inflammatory condition and response.

## Discussion

The results of this pilot study establishes the potential for biophysical profiling of immune cells as an indication of immune activation in term and preterm infants. The BLIPI system provides sensitive, quantitative measures in relation to the WBC’s activation status. The immune responses induced by the presence of inflammation were expressed in the biophysical properties of these cells, with differences noted in size and deformation. Our findings demonstrate the potential for patient classification based on this biophysical assay, with distinct biophysical profiles noted between term and preterm neonates, emphasizing the assay’s sensitivity to the potential differences in neonatal immune development and exposures. These biophysical parameters were further correlated with established clinical biomarkers of inflammation and immune responses in the infant, such as CRP, WBC counts, and I:T ratio. This correlation underscores the assay’s potential to address the current unmet need for a rapid, minimally invasive, blood-sparing and sensitive test that is capable of capturing the dynamic immune status of neonates in real-time. This ability is especially crucial as pro-inflammatory markers are linked to the development and progression of severe prematurity-related morbidities, including BPD,^19^ NEC,^8^ periventricular leukomalacia^20^ and retinopathy of prematurity.^21^ The compact and deployable system for processing clinical samples was successfully implemented in a busy tertiary-level NICU, enabling on-site measurements. To our knowledge, the test of the BLIPI system is the first instance of immune cell response profiling directly performed in the NICU.

This biophysical profiling approach would be particularly advantageous in caring for sick newborns and preterm infants, where prompt clinical management and minimization of blood loss from repeated sampling are essential. The low blood volume requirement for this test is critically important, making it particularly attractive for newborn infants whose total blood volume is only 80–100 ml/kg of body weight. This translates to about 280–350 ml for term infants and 40–50 ml for a 500 gm preterm infant. The 50 microliter requirement (1–2 drops of blood) is only 1/10 of the 500 microliter minimum requirements for CRP or full blood count. The microfluidic technology using a disposable silicone polymer test chip, also allows for rapid processing of and profiling of immune response at point-of-care, requiring only <15 min.

Even as there are no existing studies on how immune cell size or deformability differences manifest among term and preterm infants, evidence from adult cohorts has shown that an increase in size is directly linked to an increase in inflammation and activation of immune cells.^18^ Studies of immune cell biophysics has revealed that cell size, deformability, and shape are indicators of activation, metabolism, and disease.^22–25^ High-throughput single-cell mechano-phenotyping has demonstrated that activated immune cells exhibit increased deformability and size, with observed fluctuations in immune activity during activation and sepsis.^22,23,26,27^ These methods often require significant sample dilution, high-speed imaging, and extensive data processing, making it challenging to perform at point-of-care.^28,29^

The implications of successful implementation and early validation of this testing strategy are potentially vast, allowing for a direct real-time measurement of the infant’s health status and any perturbation in their clinical condition. This pilot study provides proof-of-concept for the use of biophysical measurements of immune cells to correlate with clinical conditions in neonatal care, albeit in a small group of patients. Early detection of severe conditions such as sepsis and necrotizing enterocolitis will allow the clinician to provide appropriate and timely clinical support and adjust treatment strategies to prevent the deterioration and development of poor outcomes. The ability to trend levels of inflammation in the newborn period can allow for the development of potential targeted anti-inflammatory therapies to prevent complications, especially among preterm infants.^14^ In this prospective study, only one out of eleven preterm infants was diagnosed with bacterial infection, limiting the ability to evaluate the device’s overall sensitivity, specificity, and overall diagnostic accuracy. We also acknowledge the potential differences between the biophysical profile of cord blood samples compared to peripheral blood samples from term infants. The lack of routinely performed blood samples for blood count indices and inflammatory markers (CRP) from well, term infants precluded comparisons with the biophysical profile from term infants. Bootstrapping methods described here are commonly used to simulate unknown variations, introduce noise to the model and minimise over-fitting of data. However, any real impact of the modelling requires a significantly larger clinical cohort for confirmation of these findings of the BLIPI platform. These studies would allow for the appropriate evaluation and validation of the utility of biophysical immune profiling in both term and preterm infants to enable early diagnosis and monitoring of critical clinical conditions during infancy.

## Supplementary information

Supplementary information

Supplementary video 1

Supplementary video 2

Supplementary video 3

Supplementary video 4

## Data Availability

Deidentified clinical and test data are available upon reasonable request pending data sharing agreements to ensure participant confidentiality.
