# Supplementary material for: Whole blood biophysical immune profiling of newborn infants correlates with immune responses
Source: Pediatr Res. 2025 Mar 31;98(6):2256–63. doi: 10.1038/s41390-025-03952-y (PMC12811127; doi:10.1038/s41390-025-03952-y)

Supplementary Figure S1: Immune biophysical raw histogram data based on size and deformability. The raw data is transformed into a biophysical marker feature set for as an immune signature.

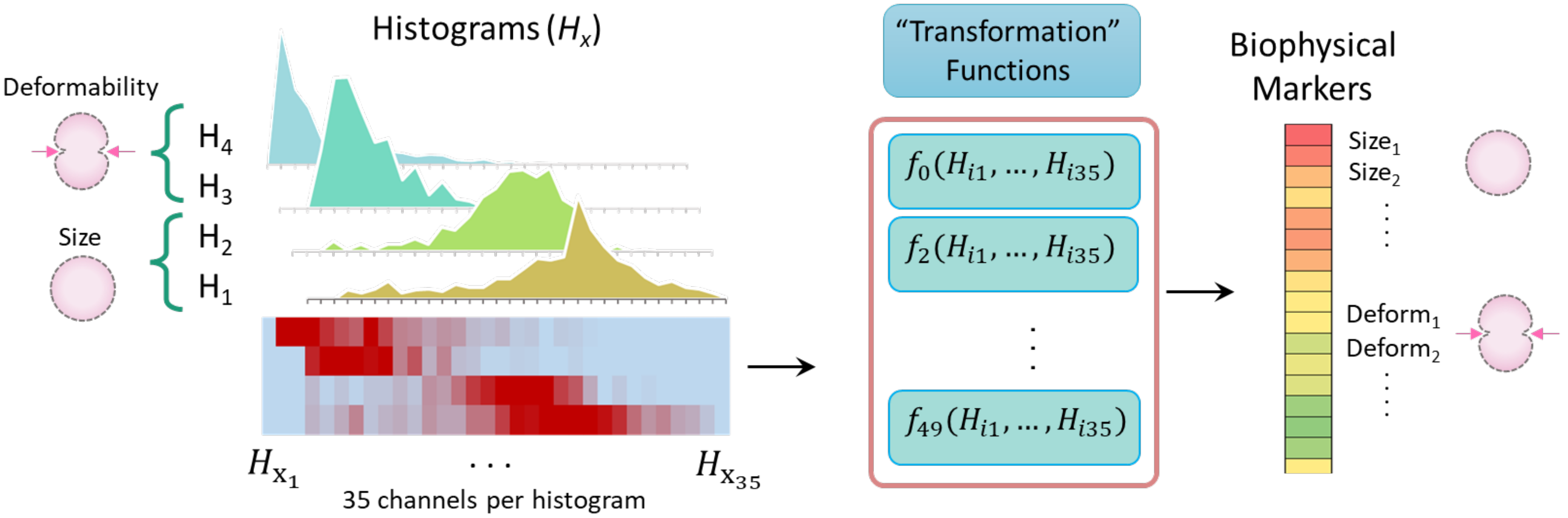

Supplementary Table S1. Transform functions of the 50 biophysical signatures

Biophysical Label	Label Description	Formula
S0	Weighted mean of H1	Weighted mean of $0 < \mathrm{i} \leq 35,\ H_{1_i}$
S1	Gated mean of H1	$(\sum_{i=7}^{35} H_{1_i} \times i)/\ \sum_{i=7}^{35} H_{1_i}$
S2	Value of H2 at histogram bin 24	$H_{2_{24}}$
S3	Mean value of H2	$(\sum_{i=0}^{35} H_{2_i} \times i)/\ \sum_{i=0}^{35} H_{2_i}$
S4	Average means of H1 and H2	$[(\sum_{i=0}^{35} H_{1_i} \times i)/\ \sum_{i=0}^{35} H_{1_i} + (\sum_{i=0}^{35} H_{2_i} \times i)/\ \sum_{i=0}^{35} H_{2_i}]\ /2$
S5	Selected gated population of H2	$\sum_{i=28}^{35} H_{2_i}$
S6	Value of H2 at histogram bin 31	$H_{2_{31}}$
S7	Value of H2 at histogram bin 28	$H_{2_{28}}$
S8	Value of H2 at histogram bin 29	$H_{2_{29}}$
S9	Value of H2 at histogram bin 30	$H_{2_{30}}$
S10	Standard deviation H2	Standard Deviation of $H_{2_{1-35}}$
D11	Value of H3 at histogram bin 2	H_{3_2}
D12	Value of H3 at histogram bin 1	H_{3_1}
S13	Selected gated population of H1	$\sum_{i=28}^{35} H_{1_i}$
D14	Ratio of selected gated population of H2 and H4	$\sum_{i=16}^{21} H_{2_i}\ /\ \sum_{i=16}^{21} H_{4_i}$
S15	Value of H2 at histogram bin 26	$H_{2_{26}}$
S16	Value of H2 at histogram bin 27	$H_{2_{27}}$
D17	Average of means of H2 and H4	$[(\sum_{i=0}^{35} H_{2_i} \times i)/\ \sum_{i=0}^{35} H_{2_i} + (\sum_{i=0}^{35} H_{4_i} \times i)/\ \sum_{i=0}^{35} H_{4_i}]\ /2$
S18	Value of H2 at histogram bin 25	$H_{2_{25}}$
S19	Gated mean of H2	$(\sum_{i=7}^{35} H_{2_i} \times i)/\ \sum_{i=7}^{35} H_{2_i}$
S20	Selected gated population of H2	$\sum_{i=22}^{27} H_{2_i}$
S21	Weighted gated population of H2	$\sum_{i=28}^{35} H_{2_i}\ /\ \sum_{i=7}^{35} H_{2_i}$
S22	Weighted mean of H2	Weighted mean of $0 < \mathrm{i} \leq 35,\ H_{2_i}$
S23	Weighted median of H2	Weighted median of $0 < \mathrm{i} \leq 35,\ H_{2_i}$
D24	Value of H4 at histogram bin 22	$H_{4_{22}}$
D25	Selected gated population of H4	$\sum_{i=16}^{21} H_{4_i}$
D26	Value of H4 at histogram bin 21	$H_{4_{21}}$
D27	Value of H4 at histogram bin 25	$H_{4_{25}}$
D28	Average of means for H3 and H4	$[(\sum_{i=0}^{35} H_{3_i} \times i)/\ \sum_{i=0}^{35} H_{3_i} + (\sum_{i=0}^{35} H_{4_i} \times i)/\ \sum_{i=0}^{35} H_{4_i}]\ /2$
D29	Value of H4 at histogram bin 23	H4-23

D30	Average of means of H1 and H3	$[(\sum_{i=0}^{35} H_{1_i} \times i)/ \sum_{i=0}^{35} H_{1_i} + (\sum_{i=0}^{35} H_{3_i} \times i)/ \sum_{i=0}^{35} H_{3_i}]/2$
D31	Mean of H3	$(\sum_{i=0}^{35} H_{3_i} \times i)/ \sum_{i=0}^{35} H_{3_i}$
D32	Value of H3 at histogram bin 28	$H_{3_{28}}$
D33	Value of H3 at histogram bin 32	$H_{3_{32}}$
D34	Weighted median of H3	Median of $H_{3_{1-35}}$
D35	Value of H3 at histogram bin 30	$H_{3_{30}}$
D36	Value of H3 at histogram bin 31	$H_{3_{31}}$
D37	Value of H3 at histogram bin 27	$H_{3_{27}}$
D38	Value of H3 at histogram bin 29	$H_{3_{29}}$
D39	Selected gated population of H3	$\sum_{i=16}^{21} H_{3_i}$
S40	Value of H2 at histogram bin 19	$H_{2_{19}}$
S41	Weighted gated population of H2	$\sum_{i=16}^{21} H_{2_i} / \sum_{i=7}^{35} H_{2_i}$
S42	Value of H2 at histogram bin 18	$H_{2_{18}}$
S43	Value of H2 at histogram bin 17	$H_{2_{17}}$
S44	Value of H2 at histogram bin 16	$H_{2_{16}}$
S45	Selected gated population of H2	$\sum_{i=10}^{15} H_{2_i}$
D46	Kullback–Leibler divergence of H3 and H4	$\sum_{i=1}^{35} H_{3_i} \log(\frac{H_{3_i}}{H_{4_i}})$
D47	Value of H3 at histogram bin 21	$H_{3_{31}}$
D48	Skew of H3	Skewness H_{3_i}
D49	Kurtosis of H3	Kurtosis H_{3_i}

Supplementary Table S2. Individual clinical characteristics and laboratory parameters of preterm cohort

Infant	Sample number	Median gestational age, weeks	Median birthweight, gm	Clinical Chorioamnionitis	Singleton	Small-for-gestational	Receipt of antenatal steroids	Age at testing, days	CRP at testing, mg/dL	WBC count, $x10^9/L$	I:T ratio
1	1	24	650	No	Yes	No	Yes	3	9.0	7.25	0.11
	2							4	9.0	7.25	0.11
2	3	30	1480	No	Yes	No	Yes	2	3.2	8.84	0.00
3	4	24	585	No	Yes	No	Yes	1	11.0	9.53	0.16
	5							6	2.1	10.13	0.04
4	6	23	625	No	Yes	No	Yes	5	18.3	26.16	0.04
	7							9	2.0	18.50	0.01
5	8	27	700	No	Yes	No	Yes	12	51.1	9.15	0.02
6	9	28	1300	No	Yes	No	Yes	5	0.0	25.19	0.03
7	10	25	750	Yes	Yes	No	Yes	33	NA	11.67	0.02
8	11	24	660	No	Yes	No	Yes	10	NA	16.5	0.02
9	12	24	590	No	Yes	No	Yes	2	16.8	39.95	0.00
	13							4	7.1	22.98	0.01
	14							23	3.5	10.70	0.02
10	15	24	750	No	Yes	No	No	6	1.8	5.65	0.23
11*	16	29	1420	Yes	Yes	No	Yes	2	99.4	6.7	0.03

ANC – absolute neutrophil count; CRP – c-reactive protein; I:T – immature : total neutrophil

* Infant with early onset sepsis

Supplementary Figure S2: Workflow for linear regression (LR) modelling using data bootstrapping to select the feature cut-off and selection. The first step is to plot the LR performances using Pearson's correlation versus the number of features used in the LR. This determines the estimated feature number cut-off. The second step involves using the RFE function in scikit-learn python library to eliminate the weakest feature and select the top 15 contributing features to the LR model. The final step is to use the 15 features to create a final Linear Regression model using a split train-test to simulate blind datasets and bootstrapping methods.

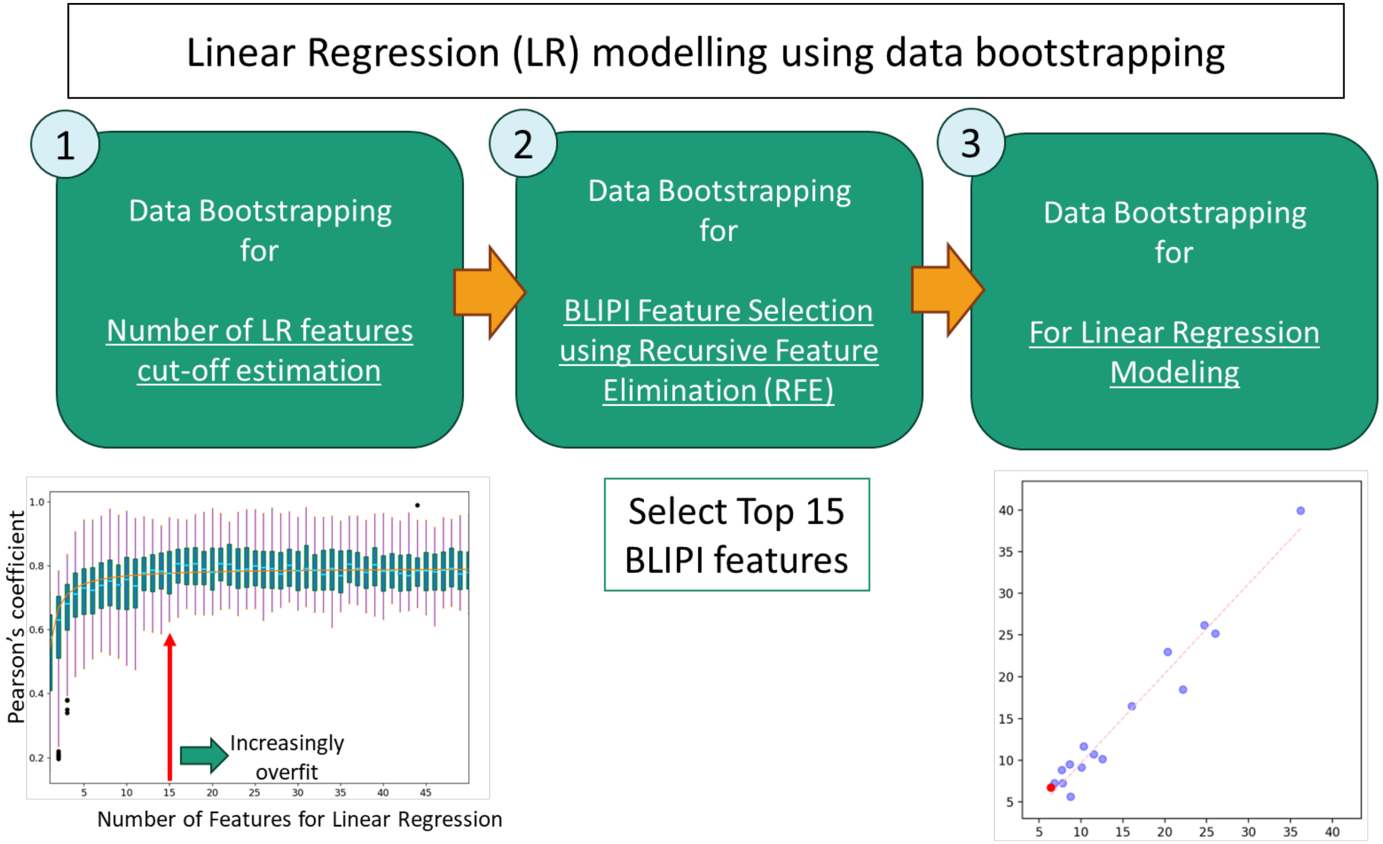

Supplementary Figure S3. The algorithm flow for selection of 15 BLIPI features for each clinical parameter (CRP, WBC counts and I:T ratio) using the Recursive Feature Elimination method.

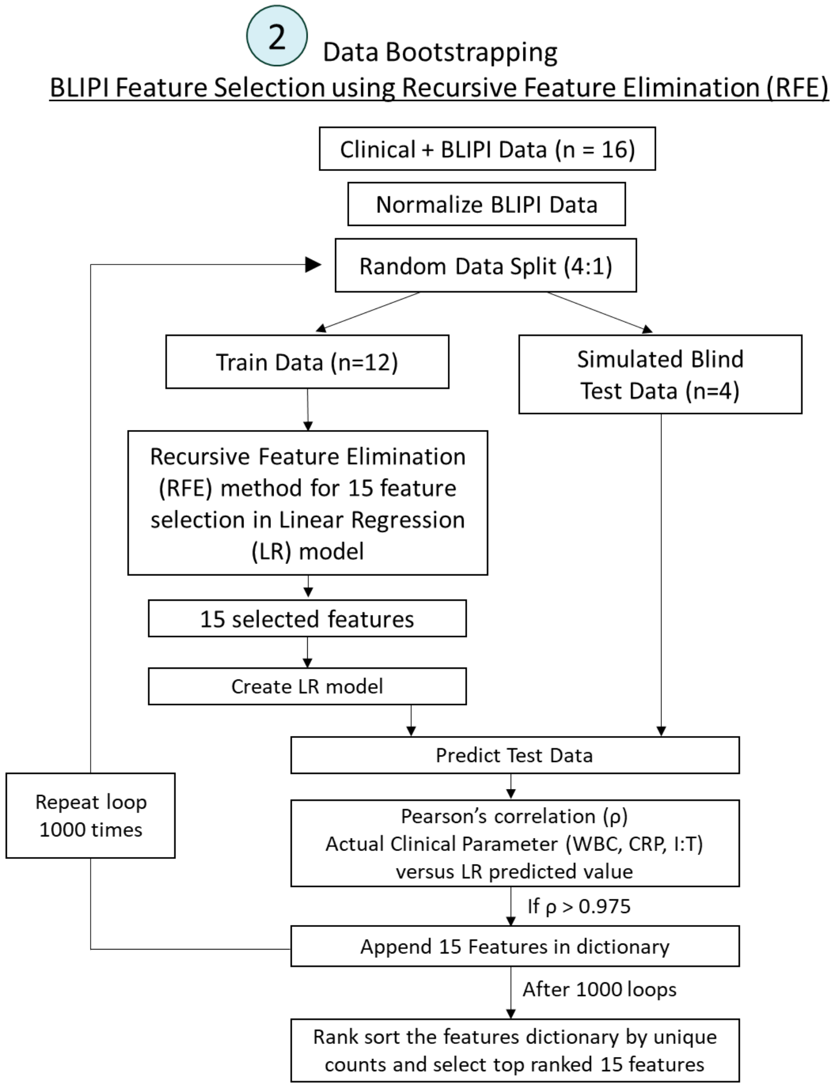

Supplementary Figure S4. Bootstrapping method for LR prediction of WBC, CRP and I:T values from BLIPI features. There are two iterations to bootstrap the data using random selecting of the data set.

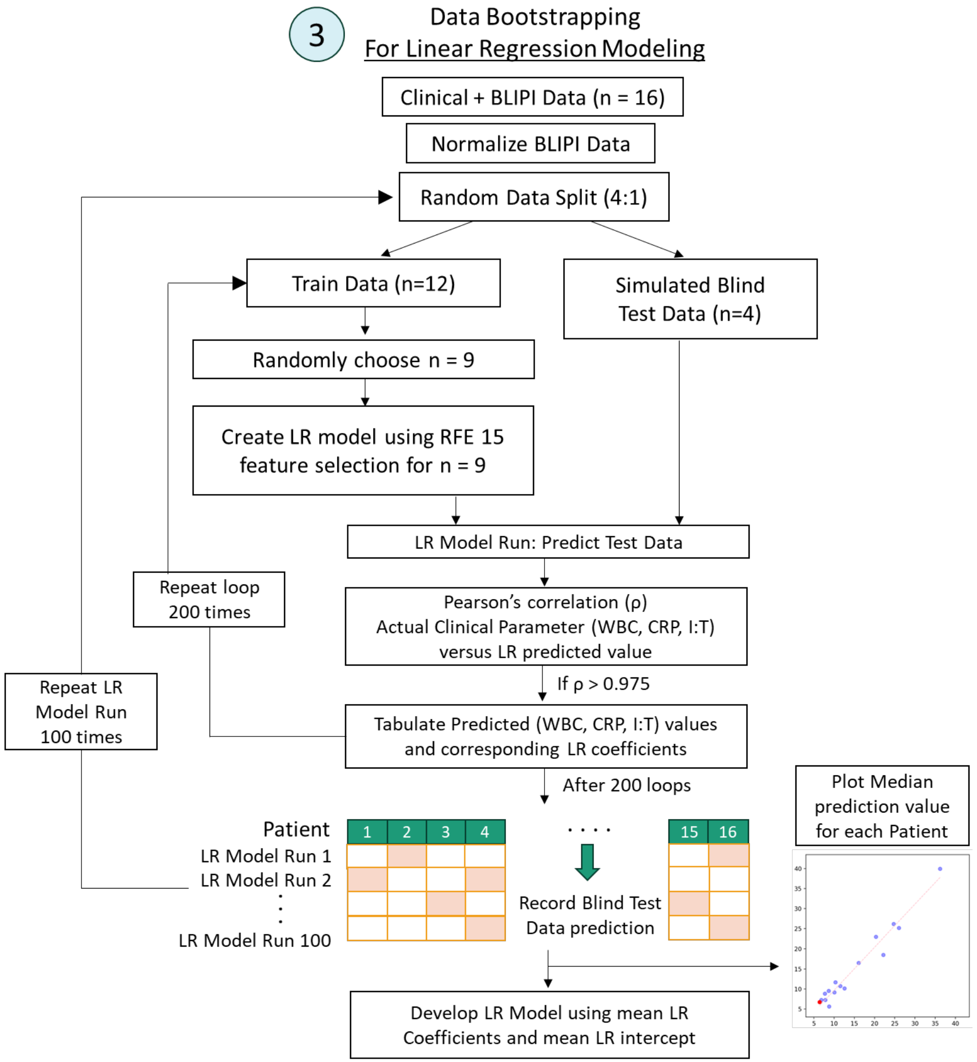

Supplementary Figure S5. Linear regression performance for CRP, WBC and I:T respectively based on the number of features used for linear regression model. The red arrow shows the cut-off feature number estimated for use in this study. Beyond 15 features, there is a tendency for overfitting.

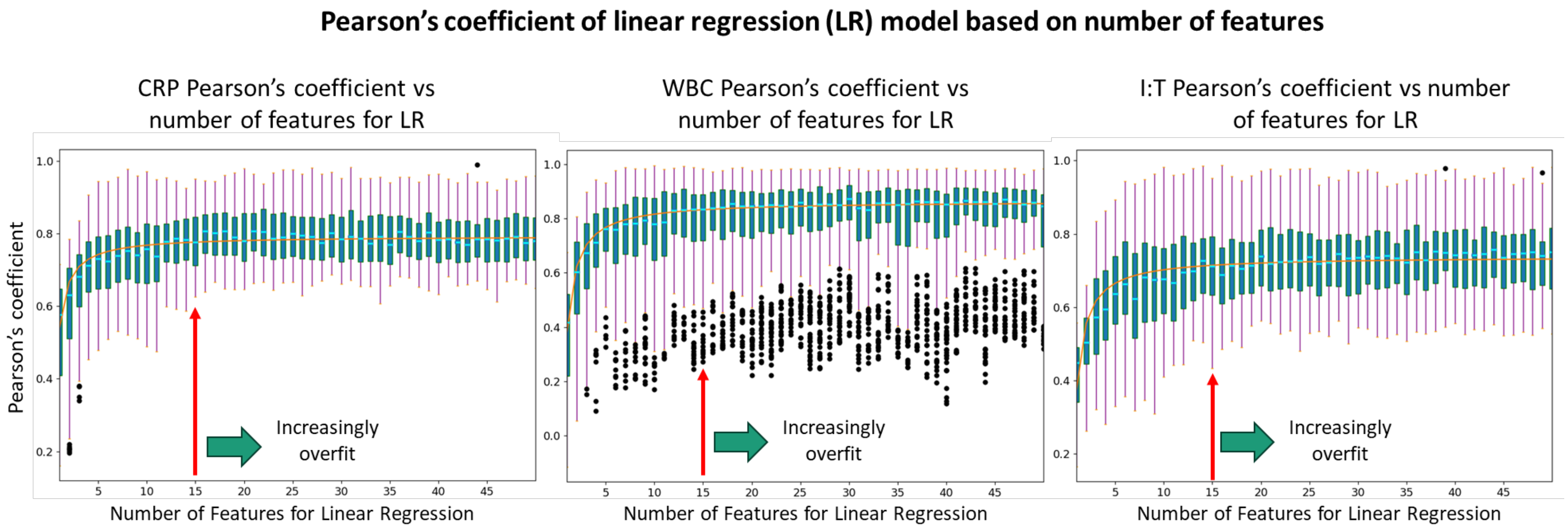

Supplement: Supplementary file 1 — Supplementary information [file 41390_2025_3952_MOESM1_ESM.pdf]
